# Synthesis and antileishmanial evaluation of some 2,3-disubstituted-4(3*H*)-quinazolinone derivatives

**DOI:** 10.1186/s13588-014-0010-1

**Published:** 2014-09-17

**Authors:** Yihenew Simegniew Birhan, Adnan Ahmed Bekhit, Ariaya Hymete

**Affiliations:** 1 Department of Chemistry, Natural and Computational Science College, Debre Markos University, Debre Markos, Ethiopia; 2 Department of Pharmaceutical Chemistry, Alexandria University, Alexandria, 21215 Egypt; 3 Department of Pharmaceutical Chemistry and Pharmacognosy, School of Pharmacy, Addis Ababa University, Addis Ababa, Ethiopia

**Keywords:** Quinazolinones, Leishmania, Antileishmanial activities

## Abstract

**Background:**

Leishmaniasis is a neglected tropical parasitic diseases affecting millions of people around the globe. Quinazolines are a group of compounds with diverse pharmacological activities. Owing to their promising antileishmanial activities, some 3-aryl-2-(substitutedstyryl)-4(3*H*)-quinazolinones were synthesized in good yields (65.2% to 86.4%).

**Results:**

The target compounds were synthesized by using cyclization, condensation, and hydrolysis reactions. The structures of the synthesized compounds were determined using elemental microanalysis, infrared (IR), and proton nuclear magnetic resonance (^1^H NMR). The *in vitro* antileishmanial activities of the synthesized compounds were evaluated using *Leishmania donovani* strain. All the synthesized compounds displayed appreciable antileishmanial activities (IC_50_ values, 0.0128 to 3.1085 μg/ml) as compared to the standard drug miltefosine (IC_50_ = 3.1911 μg/ml). (*E*)-2-(4-chlorostyryl)-3-*p*-tolyl-4(3*H*)-quinazolinone (**7**) is the compound with the most promising antileishmanial activities (IC_50_ = 0.0128 μg/ml) which is approximately 4 and 250 times more active than the standard drugs amphotericin B deoxycholate (IC_50_ = 0.0460 μg/ml) and miltefosine (IC_50_ = 3.1911 μg/ml), respectively.

**Conclusions:**

The results obtained from this investigation indicate that the synthesized and biologically evaluated quinazoline compounds showed promising antileishmanial activities and are good scaffolds for the synthesis of different antileishmanial agents.

**Electronic supplementary material:**

The online version of this article (doi:10.1186/s13588-014-0010-1) contains supplementary material, which is available to authorized users.

## Background

Leishmanisis is a neglected tropical disease resulting from infection of macrophages by obligate intracellular parasites of the genus *Leishmania*[1]-[3]. It is a public health problem in at least 88 countries with an estimated 350 million people at risk. The estimated global prevalence of all forms of the disease is 12 million. Every year, 1.5 to 2 million new cases and 70,000 deaths occur due to cutaneous leishmaniasis (CL). In addition, 500,000 new cases and 59,000 deaths from visceral leishmaniasis (VL) occur annually [4]. The number of cases of leishmaniasis is increasing globally due to *Leishmania*/HIV co-infection [5],[6], international travel, and migration of immigrants and refugees from endemic regions [7],[8].

The prophylactic treatment of leishmaniasis mainly rely on vector and reservoir control [9]-[11]. Control of reservoir host and vector is difficult due to high coast, operational difficulties, and frequent relapses in the host [12]. Although considerable effort has been made to produce vaccine candidates for the treatment of leishmaniasis, there is no vaccine against any form of human leishmaniasis yet [13]-[17].

Pentavalent antimonials (Sb^V^) have been used for the treatment of leishmania infections. Unfortunately, in many parts of the world, the parasite has become resistant to Sb^V^[18]. Treatment failure to sodium stibogluconate (SSG) is observed in Eastern Sudan [19] and in Tigray, Northern Ethiopia [20]. Recent reports showed that pentamidine also developed resistance as well as difficulties in treating patients with *Leishmania*/HIV co-infection [21].

Combination chemotherapy has improved prospects for decreasing the emergence of drug resistance, increasing activity, and reducing required doses and thereby toxic side effects. In the previous study, WR 279,396 (a topical formulation containing 15% paromomycin and 0.5% gentamicin) was found to be safe and effective against CL caused by *Leishmania major*[22]. In addition, AmBisome-paromomycin is the most cost-effective combination among miltefosine-paromomycin and AmBisome-miltefosine [23]. So far, no combination chemotherapy has been used in treatment programs, except paromomycin/SSG [24].

Tremendous quinazoline derivatives are synthesized in the past two decades, using different synthetic pathways [25]-[30], due to their diverse pharmacological activities [31]-[36] including antileishmanial activities [37]-[40]. These reports indicate that several quinazolines were synthesized and tested for their antileishmanial activities, with the aim of discovering alternative chemotherapeutic agents for the development of new antileishmanials. Promising antileishmanial activities were observed in some 4-aminoquinazoline [37], indolo[2,1-*b*]quinazoline-6,12-dione [38], and 2,3-disubstituted-4(3*H*)-quinazolinone derivatives [39],[40]. As part of the efforts to discover less toxic and more effective drug analogues for the treatment of leishmaniasis, we synthesized some 2,3-disubstituted-4(3*H*)-quinazolinones and tested their *in vitro* antileishmanial activities.

## Methods

### Chemicals and reagents

Anthranilic acid, acetic anhydride, aniline, *p*-toluidine, *o*-toluidine, acetone, dimethylsulfoxide, anhydrous zinc chloride, *p*-chlorobenzaldehyde, *p*-nitrobenzaldehyde, *p*-hydroxybenzaldehyde, chloroform, absolute ethanol, resazurin sodium salt, anhydrous petroleum ether, distilled water, iodine, HCl, and KOH were used in the study.

### Instruments and apparatuses

Melting points were determined in open capillaries using electro-thermal 9100 melting point apparatus and were uncorrected. Infrared (IR) spectra in nujol were recorded with the SHIMADZU 8400SP FT-IR spectrophotometer (Shimadzu Corporation, Nakagyo-ku, Kyoto, Japan), and proton nuclear magnetic resonance (^1^H NMR) spectral data were performed on Bruker Avance DMX400 FT-NMR spectrometer (Bruker, Billerica, MA, USA) using tetramethyl silane (TMS) as internal standard. Silica gel TLC plates of 0.25-mm thickness were used in the study.

### Experimental animals and strains

Swiss albino male mice of weight 20 to 32 g and age 6 to 8 weeks (for acute toxicity test) were obtained from Biomedical Laboratory, Department of Biology, Faculty of Science, AAU. *Leishmania donovani* isolate used in this study was obtained from Leishmania Diagnosis and Research Laboratory (LDRL) culture bank, School of Medicine, AAU.

### Culture medium and conditions

RPMI-1640, 10% heat-inactivated fetal calf serum (HIFCS), 1% penicillin-streptomycin, and 1% l-glutamine were supplied to make a complete culture medium. The *L. donovani* isolate was grown first on Novy-MacNeal-Nicolle (NNN) medium and then in tissue culture flasks containing RPMI-1640 medium supplemented with 10% HIFCS and 1% 100 IU penicillin/ml-100 μg/ml streptomycin solution at 22°C for promastigotes.

### Reference drugs

Miltefosine/hexadecylphosphocholine (AG Scientific, San Diego, CA, USA) and amphotericin B deoxyhcholate (Fungizone®, ER Squibb, Middlesex, UK) were employed as reference drugs in the *in vitro* antileishmanial activity testing of the synthesized compounds.

### Preparation of stock and working solutions

Stock solutions of 10 mg/ml of the synthesized compounds were prepared by dissolving each compound in DMSO. Stock solutions were diluted using complete RPMI to obtain aliquots of 10 μg/ml. Then, threefold serial dilution with complete RPMI gave the final six working concentrations (10, 3.33, 1.11, 0.37, 0.12, and 0.04 μg/ml) of each of the synthesized compounds. Amphotericin B deoxycholate and miltefosine, which were used as a positive control for comparison of the antileishmanial activities of the test compounds, were also made in threefold serial dilutions. All the prepared drugs were stored at −20°C and retrieved only during use [41].

### In vitro antileishmanial activity

In a 96-well microtiter plate, 100 μl of each of the seven threefold serial dilutions of synthesized compounds were added in triplicate wells. Then, 100 μl of suspension of parasites (3.0 × 10^6^ promastigotes/ml of *L. donovani*) was added in duplicate. Some of the wells contained only the parasites which served as a positive control. The media and DMSO alone acted as a negative control. The contents of the plates were then maintained in humidified atmosphere at 22°C under 5% CO_2_.

After 68 h of incubation, 10 μl of fluorochrome resazurin solution (12.5 mg dissolved in 100 ml of distilled water) was added into each well. The fluorescence intensity was measured after a total incubation period of 72 h using Victor3 Multilabel Counter (PerkinElmer, Waltham, MA, USA), at an excitation wavelength of 530 nm and emission wavelength of 590 nm [42]. The IC_50_ values were evaluated from sigmoidal dose-response curves using GraphPad Prism 5.0 software (GraphPad Software, Inc., San Diego, CA, USA).

### In vivo acute toxicity test

The oral acute toxicity of compound **7** that exhibited promising antileishmanial activity was investigated using male Swiss albino mice (approximately 20 g each) following reported methods [43]. The experimental animals were divided into six groups (containing six mice per group) and fasted overnight. Groups **1-5** received compound **7** suspended in a vehicle containing 1% gum acacia, in doses of 10, 50, 100, 200, and 300 mg/kg, respectively. The sixth group received vehicle containing 1% gum acacia (served as a control group) at a maximum dose of 1 ml/100 g of body weight by oral route. The mice were observed closely for 24 h with special attention to the first 4 h. Acute toxicity signs were checked in the test mice.

### Statistical analysis

The IC_50_ values for *in vitro* promastigotes assay of synthesized compounds were evaluated from sigmoidal dose-response curves using computer software GraphPad Prism 5.0.

## Results and discussion

### Chemistry of the synthesized compounds

Synthesis of the target compounds involved the formation of **2-5** and **10** as intermediates. It was accomplished using nucleophilic reaction, nucleophilic with ring opening and closing, condensation reaction, and hydrolysis reactions. The target compounds are synthesized in a good yield, which ranged from 65.2% to 86.4% (Table 1). All the synthesized compounds were readily soluble in DMSO and chloroform except compound **12** which is readily soluble in acetone. Spectral data (IR and ^1^H NMR) of the synthesized compounds were in full agreement with the proposed structures.

**Table 1:** **Physical constants and percent yield of the synthesized compounds**

Test compound	Molecular formula	Molecular weight (g/mol)	% yield	Melting point (°C)	***R***_f_values [CHCl_3_/C_6_H_6_(9:1)]
**6**	C_22_H_17_ClN_2_O	360.85	68.3	201 to 203	0.520
**7**	C_23_H_19_ClN_2_O	374.87	65.2	189 to 191	0.577
**8**	C_23_H_18_N_3_O_3_	384.41	74.8	214 to 216	0.422
**9**	C_23_H_18_N_3_O_3_	384.41	76.2	235 to 237	0.642
**11**	C_26_H_24_N_2_O_4_	428.49	86.4	151 to 153	0.781
**12**	C_22_H_18_N_2_O_2_	342.40	80.3	298 to 300	0.524
**13**	C_24_H_22_N_2_O_3_	386.45	82.2	196 to 198	0.711

### Biological activity testing results

#### In vitro antileishmanial activity of the synthesized compounds

The antipromastigote activities of the synthesized compounds and the standard antileishmanial drugs (amphotericin B deoxycholate and miltefosine) were evaluated using the clinical isolate of *L. donovani* strain. The IC_50_ of the synthesized and reference drugs were evaluated from fluorescence characteristic of AlamarBlue® (resazurin) (Trek Diagnostic Systems, Inc., Cleveland, OH, USA) which is soluble, stable in culture medium, non-toxic to cells, and does not affect the secretary abilities of cells [44]. The test works as a cell viability and proliferation indicator through the conversion of resazurin to resorufin via reduction. The amount of fluorescence produced is proportional to the number of living cells [45],[46].

The quinazolinone derivatives synthesized were shown to have good antileishmanial activity which was in line with the previous reports [37]-[40]. All the tested compounds exhibited better antileishmanial activity than the standard drug miltefosine as shown in Table 2. Among them, compound **7** was found to have a very promising antileishmanial activity with an IC_50_ value of 0.0128 μg/ml which was 250 times superior than miltefosine (3.1911 μg/ml). Compounds **8** and **11** were 30 times more active than miltefosine. Compounds **6** and **12** were 10 times and twice more active than miltefosine, respectively. Compounds **9** and **13** were as active as miltefosine.

**Table 2:** **Antipromastigote activity (IC**_**50**_ **) of the synthesized compounds**

Test compounds	IC_50_values (μg/ml)	IC_50_values (ng/ml)
**6**	0.3014	301.40
**7**	0.0128	12.80
**8**	0.1085	108.50
**9**	2.7017	2,701.70
**11**	0.1086	108.60
**12**	1.6472	1,647.20
**13**	3.1085	3,108.50
Miltefosine	3.1911	3,191.10
Amphotericin B	0.0460	46.00

IC_50_: effective concentration required to achieve 50% growth inhibition (in μg/ml).

All the synthesized compounds except compound **7** displayed weak antileishmanial activities as compared to amphotericin B deoxycholate. Better antipromastigote activity was observed for (*E*)-2-(4-chlorostyryl)-3-*p*-tolyl-4(3*H*)-quinazolinone (**7**) with an IC_50_ value of 0.0128 μg/ml which is four times higher than the standard drug amphotericin B deoxycholate with an IC_50_ value of 0.0128 μg/ml. (*E*)-2-(4-chlorostyryl)-3-*p*-tolyl-4(3*H*)-quinazolinone (**7**) was found to be 4 times more active than amphotericin B deoxycholate and 250 times more active than miltefosine.

#### Oral acute toxicity study

Compound (*E*)-2-(4-chlorostyryl)-3-*p*-tolyl-4(*3H*)-quinazolinone (**7**) was observed to be devoid of any inherent acute toxicities at a maximum dose of 300 mg/kg.

## Experimental

### Synthesis of target compounds

**Scheme 1:**
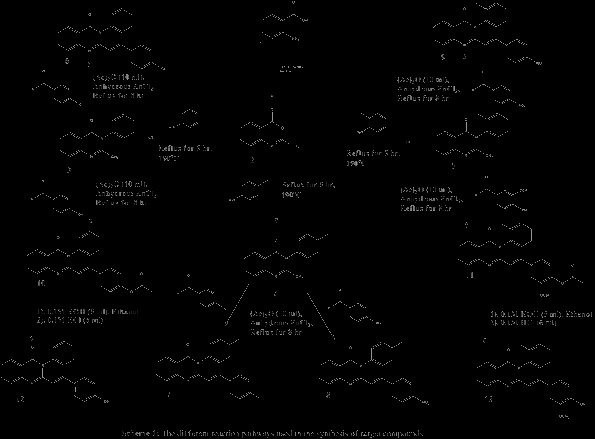
**Synthesis of 3-aryl-2-(substitutedstyryl)-4(3** ***H*** **)-quinazolinones using acetanthranil (2-methyl-3,1-benzoxazin-4-one (2)) and 3-aryl-2-methyl-4(3** ***H*** **)-quinazolinones (3-5) as intermediates.**

The synthesis of target compounds, 3-aryl-2-(substitutedstyryl)-4(3*H*)-quinazolinones (**6-9** and **11-13**), was achieved using cyclization, condensation, and hydrolysis reactions. It involved the synthesis of acetanthranil (2-methyl-3,1-benzoxazin-4-one (**2**)) and 3-aryl-2-methyl-4(3*H*)-quinazolinones (**3-5**) as intermediates (Scheme 1). The details of each reactions and reaction conditions, the summarized characteristic stretching and bending IR vibration frequencies, the elemental microanalysis, and the ^1^H NMR chemical shift data for each of the synthesized target compounds are given below.

#### General procedure for the synthesis of 2-methyl-3,1-benzoxazin-4-one (2)

A solution of anthranillic acid (**1**) (10 g, 0.073 mol) in acetic anhydride (25 ml) was heated under reflux for 1 h. The precipitate formed on cooling was filtered and the excess acetic anhydride was washed with anhydrous petroleum ether, where upon a solid mass is obtained. This solid mass (**2**), without purification, was used for subsequent reaction [47].

#### General procedure for the synthesis of 3-aryl-2-methyl-4(3H)-quinazolinones (3-5)

A mixture of 2-methyl-3,1-benzoxazin-4-one (**2**) (3 g, 0.017 mol) and equimolar amounts of aromatic amines (aniline, *p*-toluidine, and *o*-toluidine, respectively) was heated under reflux at 190°C for 5 h. The dark sticky mass formed were cooled and recrystallized from ethanol to yield compounds **3-5**, respectively [48].

#### General procedure for the synthesis of 3-aryl-2-(4-chlorostyryl)-4(3H)-quinazolinones (6 and 7)

To a solution of **3** or **4** (0.5 g each) in acetic anhydride (10 ml), an equimolar amount of *p*-chlorobenzaldehyde was added in the presence of 10 mg of anhydrous zinc chloride as a catalyst. The reaction mixture was heated under reflux for 8 h, cooled, and poured into ice-cooled water. The solid products formed (**6** or **7**) were filtered, dried, and recrystallized from chloroform/ethanol (2:1) [49].

#### E)-2-(4-chlorostyryl)-3-phenylquinazolin-4(3H)-one (6)

IR (Nujol) (cm^−1^): 1,682 (C = O), 1,597 (C = N), and 1,224 (C-Cl). ^1^H NMR (CDCl_3_) *δ* (ppm): 6.33 (*d*, 1H, *J* = 15.49 Hz, vinyl-C_2_ H), 7.23 (*d*, 2H, *J* = 8.53 Hz, 4-chlorophenyl C_3,5_ H), 7.28 (*d*, 2H, *J* = 8.47 Hz, 4-chlorophenyl C_2,6_ H), 7.34 (*d*, 2H, *J* = 6.82 Hz, phenyl C_2,6_ H), 7.45 to 7.49 (*m*, 1H, quina-C_6_ H), 7.58 to 7.63 (*m*, 3H, phenyl C_3,4,5_ H), 7.75 to 7.79 (*m*, 2H, quina-C_7,8_ H), 7.91 (*d*, 1H, *J* = 15.47 Hz, vinyl-C_1_ H), 8.29 (*d*, 1H, *J* = 7.95 Hz, quina-C_5_). Anal. calcd. for C_22_H_17_ClN_2_O: C, 73.23; H, 4.75; Cl, 9.83; N, 7.76. Found: C, 73.64; H, 4.92; Cl, 10.22; N, 7.54.

#### E)-2-(4-chlorostyryl)-3-p-tolylquinazolin-4(3H)-one (7)

IR (Nujol) (cm^−1^): 1,682 (C = O), 1,597 (C = N), and 1,224 (C-Cl). ^1^H NMR (CDCl_3_) *δ* (ppm): 2.5 (*s*, 3H, *p-* tolyl CH_3_), 6.42 (*d*, 1H, *J* = 15.70 Hz, vinyl-C_2_ H), 7.21 (*d*, 2H, 4-chlorophenyl C_3,5_ H), 7.26 to 7.32 (*m*, 4H, *p*-tolyl C_2,3,5,6_ H), 7.40 (*d*, 2H, 4-chlorophenyl C_2,6_ H), 7.47 to 7.51 (*m*, 1H, quina-C_6_ H), 7.79 to 7.83 (*m*, 2H, quina-C_7,8_ H), 7.93 (*d*, 1H, vinyl-C_1_ H), 8.32 (*d*, 1H, quina-C_5_ H). Anal. calcd. for C_23_H_19_ClN_2_O: C, 73.69; H, 5.12; Cl, 9.46; N, 7.47. Found: C, 73.98; H, 5.38; Cl, 9.35; N, 7.21.

#### General procedure for the synthesis of 3-aryl-2-(4-nitrostyryl)-4(3H)-quinazolinones (8 and 9)

To a solution of **4** or **5** (0.5 g each) in acetic anhydride (10 ml), an equimolar amount of *p*-nitrobenzaldehyde was added in the presence of 10 mg of anhydrous zinc chloride as a catalyst. The reaction mixture was heated under reflux for 8 h, cooled, and poured into ice-cooled water. The solid products formed (**8** or **9**) were filtered, dried, and recrystallized from chloroform/ethanol (2:1) [49].

#### E)-2-(4-nitrostyryl)-3-p-tolylquinazolin-4(3H)-one (8)

IR (Nujol) (cm^−1^): 1,684 (C = O), 1,593 (C = N), 1,556 and 1,377 (NO_2_). ^1^H NMR (CDCl_3_) *δ* (ppm): 2.5 (*s*, 3H, *p-* tolyl CH_3_), 6.56 (*d*, 1H, *J* = 15.52 Hz, vinyl-C_2_ H), 7.21 (*d*, 2H, *J* = 8.19 Hz, *p-* tolyl C_3,5_ H), 7.41 (*d*, 2H, *J* = 7.97 Hz, *p*-tolyl C_2,6_ H), 7.46 to 7.53 (*m*, 3H, 4-nitrophenyl C_2,6_ and quina-C_6_), 7.77 to 7.81 (*m*, 2H, quina-C_7,8_), 8.00 (*d*, 1H, *J* = 15.52 Hz, vinyl-C_1_), 8.19 (*d*, 2H, *J* = 8.74 Hz, 4-nitrophenyl C_3,5_), 8.30 (*d*, 1H, *J* = 8.01 Hz, quina-C_5_). Anal. calcd. for C_23_H_18_N_3_O_3_: C, 71.86; H, 4.72; N, 10.93. Found: C, 72.12; H, 4.35; N, 11.10.

#### E)-2-(4-nitrostyryl)-3-o-tolylquinazolin-4(3H)-one (9)

IR (Nujol) (cm^−1^): 1,682 (C = O), 1,593 (C = N), 1,556 and 1,377 (NO_2_). ^1^H NMR (CDCl_3_) *δ* (ppm): 2.17 (*s*, 3H, *o*-tolyl CH_3_), 6.47 (*d*, 1H, *J* = 15.66 Hz, vinyl-C_2_ H), 7.25 (*d*, 1H, *J* = 7.91 Hz, *o*-tolyl C_3_ H), 7.44 to 7.46 (*m*, 3H, 4-nitrophenyl C_2,6_ and *o*-tolyl C_6_ H), 7.47 to 7.58 (*m*, 3H, *o*-tolyl C_4,5_ and quina-C_6_ H), 7.82 to 7.89 (*m*, 2H, quina-C_7,8_ H), 8.05 (*d*, 1H, *J* = 15.56 Hz, vinyl-C_1_ H), 8.19 (*d*, 2H, *J* = 8.73 Hz, 4-nitrophenyl C_4,6_ H), 8.36 (*d*, 1H, *J* = 8.25 Hz, quina-C_5_ H). Anal. calcd. for C_23_H_18_N_3_O_3_: C, 71.86; H, 4.72; N, 10.93. Found: C, 71.68; H, 4.93; N, 11.24.

#### General procedure for the synthesis of 3-aryl-2-(4-acetylatedstyryl)-4(3H)-quinazolinones (10)

To a solution of **3** (0.5 g) in acetic anhydride (10 ml), an equimolar amount of *p*-hydroxybenzaldehyde was added. Anhydrous zinc chloride (10 mg) is added as a catalyst. The reaction mixture is heated under reflux for 8 h, cooled, and poured into ice-cooled water. The solid product formed (**10**) was filtered, dried, and recrystallized from ethanol [49].

#### General procedure for the synthesis of 3-aryl-2-(4-acetylatedstyryl)-4(3H)-quinazolinones (11)

To a solution of **5** (0.5 g) in acetic anhydride (10 ml), an equimolar amount of vanillin was added. Anhydrous zinc chloride (10 mg) is added as a catalyst. The reaction mixture is heated under reflux for 8 h, cooled, and poured into ice-cooled water. The solid product (**11**) was filtered, dried, and recrystallized from ethanol [49].

#### 1E)-2-[-3,4-dihydro-3-(2-methylphenyl)-4-oxoquinazoline-2-yl)]vinyl}-2-methoxyphenyl acetate (11)

IR (Nujol) (cm^−1^): 1,761 (C = O), 1,682 (C = O), 1,634 (C = N), 1,260 and 1,149 (C-O-C). ^1^H NMR (CDCl_3_) *δ* (ppm): 2.15 (*s*, 3H, phenylacetate CH_3_), 2.33 (*s*, 3H, *o*-tolyl CH_3_), 3.80 (*s*, 3H, methoxy -O-CH_3_), 6.27 (*d*, 1H, *J* = 15.44 Hz, vinyl-C_2_ H), 6.88 to 6.93 (*m*, 2H, 2-methoxyphenyl C_3,5_ H), 6.98 (*d*, 1H, *J* = 8.12 Hz, 2-methoxyphenyl C_6_ H), 7.24 (*d*, 1H, *J* = 7.52 Hz, *o*-tolyl C_3_ H), 7.42 to 7.53 (*m*, 4H, *o*-tolyl C_4,5,6_ H and quina-C_6_ H), 7.82 to 7.83 (*m*, 2H, quina-C_7,8_ H), 7.96 (*d*, 1H, *J* = 15.48 Hz, vinyl-C_1_ H), 8.34 (*d*, 1H, *J* = 7.88 Hz, quina-C_5_ H). Anal. calcd. for C_26_H_24_N_2_O_4_: C, 72.88; H, 5.65; N, 6.54. Found: C, 73.11; H, 5.89; N, 6.42.

#### General procedure for the synthesis of 3-aryl-2-(4-deacetylatedstyryl)-4(3H)-quinazolinones (12 and 13)

Subsequent treatment of **10** and **11** with 0.1 M alcoholic KOH (5 ml) in the presence of ethanol followed by 0.1 M HCl (6 ml) gave the corresponding 4-hydroxyl containing compounds **12** and **13** after recrystallization from ethanol [49].

#### E)-2-(4-hydroxystyryl)-3-phenylquinazolin-4(3H)-one (12)

IR (Nujol) (cm^−1^): 3,290 (OH), 1,652 (C = O), and 1,604 (C = N). ^1^H NMR (acetone-d_6_) *δ* (ppm): 6.24 (*d*, 1H, *J* = 15.39 Hz, vinyl-C_2_ H), 6.80 (*d*, 2H, *J* = 8.64 Hz, 4-hydroxyphenyl C_3,5_ H), 7.24 (*d*, 2H, *J* = 8.62 Hz, 4-hydroxyphenyl C_2,6_ H), 7.46 to 7.50 (*m*, 3H, phenyl C_3,4,5_ H), 7.60 to 7.66 (*m*, 3H, quina-C_6_, phenyl C_2,6_ H), 7.73 (*d*, 1H, *J* = 8.07 Hz, quina-C_8_ H), 7.81 to 7.85 (*m*, 1H, quina-C_7_ H), 7.92 (*d*, 1H, *J* = 15.43 Hz, vinyl-C_1_ H), 8.02 (*s*, 1H, 4-hydroxyphenyl -OH), 8.17 (*d*, 1H, *J* = 9.18 Hz, quina-C_5_ H). Anal. calcd. for C_22_H_18_N_2_O_2_: C, 7.17; H, 5.23; N, 8.18. Found: C, 76.86; H, 5.02; N, 8.38.

#### E)-2-(4-hydroxy-3-methoxystyryl)-3-o-tolylquinazolin-4(3H)-one (13)

IR (Nujol) (cm^−1^): 3,400 (OH), 1,683 (C = O), 1,634 (C = N), 1,211 and 1,148 (C-O-C). ^1^H NMR (CDCl_3_) *δ* (ppm): 2.15 (*s*, 3H, *o*-tolyl CH_3_), 3.80 (*s*, 3H, 4-hydroxy-2-methoxyphenyl -O-CH_3_), 6.10 (*s*, 1H, 4-hydroxy-2-methoxyphenyl -OH), 6.27 (*d*, 1H, *J* = 15.44 Hz, vinyl-C_2_ H), 6.88 to 6.93 (*m*, 2H, 4-hydroxy-2-methoxyphenyl C_3,5_ H), 6.98 (*d*, 1H, *J* = 8.12 Hz, 4-hydroxy-2-methoxyphenyl C_6_ H), 7.24 (*d*, 1H, *J* = 7.52 Hz, *o*-tolyl C_3_ H), 7.42 to 7.53 (*m*, 4H, *o*-tolyl C_4,5,6_ H and quina-C_6_ H), 7.82 (*m*, 2H, quina-C_7,8_ H), 7.96 (*d*, 1H, *J* = 15.48 Hz, vinyl-C_1_ H), 8.34 (*d*, 1H, *J* = 7.884 Hz, quina-C_5_ H). Anal. calcd. for C_24_H_22_N_2_O_3_: C, 74.59; H, 5.74; N, 7.23. Found: C, 74.28; H, 5.96; N, 7.56.

## Conclusions

Some 3-aryl-2-(substitutedstyryl)-4(3*H*)-quinazolinone derivatives were synthesized and tested for their antileishamanial activities. Most of the synthesized compounds displayed better antileishmanial activities as compared to the standard drug miltefosine and lower antileishmanial activity as compared to amphotericin B deoxycholate except (*E*)-2-(4-chlorostyryl)-3-*p*-tolyl-4(3*H*)-quinazolinone (**7**). Compound **7** showed pronounced antileishmanial activities as compared to miltefosine and amphotericin B deoxycholate. Thus, 2,3-disubstituted-4(3*H*)-quinazolinones containing an aromatic substitution at 3-position and substituted styryl moiety at 2-position represent a promising matrix for the development of antileishmanial agents.

## Authors’ original submitted files for images

Below are the links to the authors’ original submitted files for images. Authors’ original file for figure 1 Authors’ original file for figure 2
