# Supplementary figures and images for: Synthesis and antileishmanial evaluation of some 2,3-disubstituted-4(3H)-quinazolinone derivatives
Source: Org Med Chem Lett. 2014 Sep 17;4:10. doi: 10.1186/s13588-014-0010-1 (PMC4970432; doi:10.1186/s13588-014-0010-1)

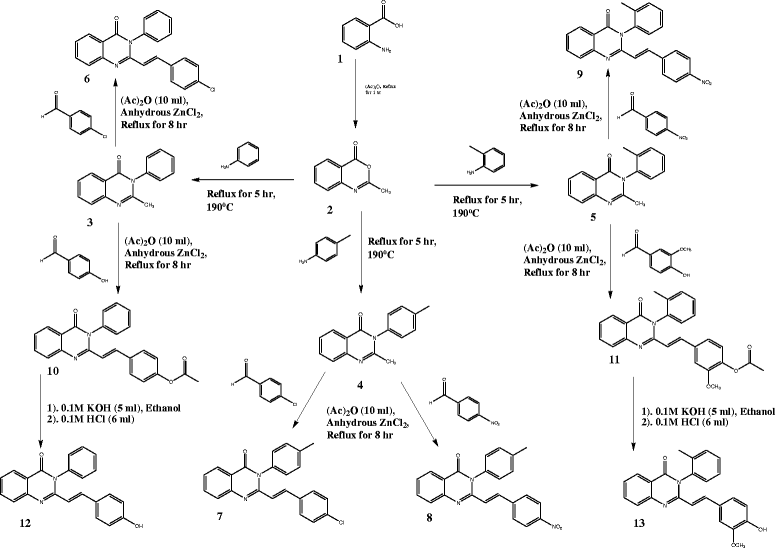

Supplement: Supplementary file 1 — Authors’ original file for figure 1 [file 13588_2014_10_MOESM1_ESM.gif]
