# Supplementary material for: Synthesis and antileishmanial evaluation of some 2,3-disubstituted-4(3H)-quinazolinone derivatives
Source: Org Med Chem Lett. 2014 Sep 17;4:10. doi: 10.1186/s13588-014-0010-1 (PMC4970432; doi:10.1186/s13588-014-0010-1)
Supplement: Supplementary file 2 — Authors’ original file for figure 2 [file 13588_2014_10_MOESM2_ESM.docx]

Table 2: Antipromastigote activity (IC_50_) of the synthesized compounds*.

| ***Test compounds*** | ***IC_50_ values (µg/ml)*** | ***IC_50_ values (ng/ml)*** |
| --- | --- | --- |
| **6** | 0.3014 | 301.40 |
| **7** | 0.0128 | 12.80 |
| **8** | 0.1085 | 108.50 |
| **9** | 2.7017 | 2701.70 |
| **11** | 0.1086 | 108.60 |
| **12** | 1.6472 | 1647.20 |
| **13** | 3.1085 | 3108.50 |
| **Miltefosine** | 3.1911 | 3191.10 |
| **Amphotericin B** | 0.0460 | 46.00 |

*IC_50_: effective concentration required to achieve 50% growth inhibition in µg/ml.
